# Supplementary material for: Selenocyanate derived Se-incorporation into the nitrogenase Fe protein cluster
Source: eLife. 2022 Jul 29;11:e79311. doi: 10.7554/eLife.79311 (PMC9462850; doi:10.7554/eLife.79311)
Supplement: Supplementary file 1. [file elife-79311-supp1.docx]

Comparison of B-factors for cluster modeled as exclusively FeS vs FeSe forms.

| All S Cluster Form | | All Se Cluster Form | |
| --- | --- | --- | --- |
| R_work_ | 16.87 | R_work_ | 16.75 |
| R_free_ | 19.28 | R_free_ | 19.02 |
| Atom | **B-factor** | **Atom** | **B-factor** |
| S3 | 11.07 | Se3 | 19.90 |
| S4 | 11.40 | Se4 | 20.18 |
| Fe1 | 16.02 | Fe1 | 15.53 |
| Fe2 | 16.41 | Fe2 | 15.89 |
