# Supplementary material for: Selenocyanate derived Se-incorporation into the nitrogenase Fe protein cluster
Source: eLife. 2022 Jul 29;11:e79311. doi: 10.7554/eLife.79311 (PMC9462850; doi:10.7554/eLife.79311)
Supplement: Supplementary file 2. [file elife-79311-supp2.docx]

B-factor analysis of cluster atoms in Se-free Fe protein crystal structures.

| PDB ID | 7TPW | 7TPX | 7TPY | 7TPZ |
| --- | --- | --- | --- | --- |
| Brief Description Reaction Conditions / Crystal | Se-free control crystal for B-factor analysis | Se-free control crystal for B-factor analysis | No nucleotide, MoFe protein, or ATP regeneration components; MgADP added during work up | ATP and ATP regeneration system replaced with MgADP |
| *Refinement Statistics Brief Overview (for full details, see Table S3)* | | | | |
| **Resolution [Å] (energy [eV])** | 1.18 (12668) | 1.35 (12668) | 1.48 (12668) | 1.71 (12668) |
| R_work_ | 14.12 | 17.63 | 17.29 | 17.12 |
| R_free_ | 15.82 | 19.49 | 19.35 | 20.53 |
| B-factor (overall) | 19.09 | 22.09 | 25.25 | 28.07 |
| *B-Factors (Å^2^)* | | | | |
| Fe1 (302) | 12.10 | 16.69 | 19.96 | 21.13 |
| Fe2 (304) | 12.09 | 16.96 | 20.10 | 21.83 |
| **Fe_avg_** | **12.10** | **16.83** | **20.03** | **21.48** |
| S3 (301) | 12.75 | 17.27 | 20.47 | 21.89 |
| S4 (303) | 12.02 | 16.59 | 19.54 | 20.66 |
| **S_avg_** | **12.39** | **16.93** | **20.01** | **21.28** |
| **Fe/S Avg Difference** | **0.29** | **0.10** | **0.02** | **0.20** |
