# Supplementary material for: Selenocyanate derived Se-incorporation into the nitrogenase Fe protein cluster
Source: eLife. 2022 Jul 29;11:e79311. doi: 10.7554/eLife.79311 (PMC9462850; doi:10.7554/eLife.79311)
Supplement: Supplementary file 3. [file elife-79311-supp3.docx]

Summary of crystallographically determined Se occupancies for KSeCN derived Se-incorporation at the Fe protein cluster under various conditions. The occupancies were determined in triplicate^§^ by analyzing three crystals prepared from a specified set of reaction conditions.

| **Entry** | **Brief Description of reaction conditions*** | **X3 Occupancy**  **individual refined occupancies**  **(average + standard deviation)** | **X4 Occupancy**  **individual refined occupancies**  **(average + standard deviation)** | |
| --- | --- | --- | --- | --- |
| **1** | 22 mM KSeCN, w/ MoFe protein | 0.51, 0.39, 0.62  (0.51 ± 0.09) | 0.45, 0.35, 0.48  (0.43 ± 0.06) | |
| **2** | 22 mM KSeCN | 0.59, 0.57, 0.54, 0.63  (0.58 ± 0.03) | 0.38, 0.36, 0.32, 0.45  (0.38 ± 0.05) | |
| **3** | 11 mM KSeCN | 0.08, 0.09, 0.04  (0.07 ± 0.02) | 0.02, 0.09, 0.07  (0.06 ± 0.03) | |
| **4** | 1 mM KSeCN | 0, 0, 0.05  (0.02 ± 0.01) | 0, 0, 0.02  (0.02 ± 0.01) | |
| ^§^With the exception of entry 2 for which four crystals were analyzed. | | | |  |
