# Supplementary material for: Selenocyanate derived Se-incorporation into the nitrogenase Fe protein cluster
Source: eLife. 2022 Jul 29;11:e79311. doi: 10.7554/eLife.79311 (PMC9462850; doi:10.7554/eLife.79311)
Supplement: Supplementary file 4. [file elife-79311-supp4.docx]

Data collection and refinement statistics for *Se-free* Fe protein crystals from various control reactions. Values in parentheses represent the highest resolution shell.

| ***Data Processing Statistics*** | | | | |
| --- | --- | --- | --- | --- |
| PDB ID | **7TPW** | **7TPX** | **7TPY**  No nucleotide during reaction, MgADP added for crystallization | **7TPZ**  MgADP control |
| Wavelength (Å) | 12668 | 12668 | 12668 | 12668 |
| Resolution range (Å) | 45.59 - 1.18  (1.19 - 1.18) | 45.59 - 1.35  (1.37 - 1.35) | 45.48 - 1.48  (1.51 - 1.48) | 45.48 - 1.71  (1.74 - 1.71) |
| Space group | P22_1_2_1_ | P22_1_2_1_ | P22_1_2_1_ | P22_1_2_1_ |
| a, b, c (Å) | 45.57 74.53 74.98 | 45.59 74.26 74.45 | 45.44 73.73 74.54 | 45.52 73.71 74.67 |
| α, β, γ (˚) | 90 90 90 | 90 90 90 | 90 90 90 | 90 90 90 |
| Unique reflections | 80169 (2524) | 56156 (2722) | 42502 (2147) | 27863 (1536) |
| Multiplicity | 11.9 (5.4) | 12.9 (11.3) | 13.1 (13.0) | 13.0 (12.9) |
| Completeness (%) | 94.9 (59.4) | 99.8 (99.6) | 99.9 (99.7) | 99.8 (98.5) |
| I/σ(I) | 16.7 (2.0) | 17.2 (1.9) | 16.3 (1.8) | 19.7 (2.0) |
| Wilson B-factor | 11.99 | 14.27 | 17.92 | 20.82 |
| R_merge_ | 0.074 (0.627) | 0.072 (1.067) | 0.079 (1.677) | 0.076 (1.273) |
| R_p.i.m._ | 0.030 (0.435) | 0.029 (0.465) | 0.032 (0.699) | 0.031 (0.526) |
| CC_1/2_ | 0.999 (0.797) | 0.999 (0.907) | 0.999 (0.835) | 1.00 (0.818) |
| ***Data Refinement Statistics*** | | | | |
| Resolution range (Å) | 38.94 - 1.18  (1.19 - 1.18) | 45.59 - 1.35  (1.37 - 1.35) | 37.27 - 1.48  (1.50 - 1.48) | 37.33 - 1.71  (1.74 - 1.71) |
| R_work_ | 0.1412 (0.2874) | 0.1763 (0.3432) | 0.1729 (0.3070) | 0.1712 (0.2826) |
| R_free_ | 0.1582 (0.3428) | 0.1949 (0.3354) | 0.1935 (0.3013) | 0.2053 (0.3809) |
| RMS(bonds) (Å) | 0.007 | 0.006 | 0.006 | 0.008 |
| RMS(angles) (°) | 1.11 | 1.03 | 1.07 | 1.10 |
| Ramachandran favored (%) | 97.81 | 98.16 | 97.78 | 97.41 |
| Ramachandran allowed (%) | 1.82 | 1.47 | 2.22 | 2.59 |
| Ramachandran outliers (%) | 0.36 | 0.37 | 0 | 0 |
| Rotamer outliers (%) | 0.44 | 0.45 | 0.88 | 0 |
| Average B-factor | 19.09 | 22.09 | 25.25 | 28.07 |
