# Supplementary material for: Selenocyanate derived Se-incorporation into the nitrogenase Fe protein cluster
Source: eLife. 2022 Jul 29;11:e79311. doi: 10.7554/eLife.79311 (PMC9462850; doi:10.7554/eLife.79311)
Supplement: Supplementary file 5. [file elife-79311-supp5.docx]

Data collection and refinement statistics for Se-incorporated Fe protein crystals derived from *22 mM* KSeCN reaction in the *presence* of MoFe protein. Values in parentheses represent the highest resolution shell.

| *Data Processing Statistics* | | | |
| --- | --- | --- | --- |
| PDB ID | **7T4H** | **7TQ0** | **7TQ9** |
| Wavelength (Å) | 12668 | 12668 | 12750 |
| Resolution range (Å) | 45.79 - 1.51  (1.53 - 1.51) | 45.94 - 1.81  (1.85 - 1.81) | 45.89 - 1.6  (1.63 - 1.60) |
| Space group | P22_1_2_1_ | P22_1_2_1_ | P22_1_2_1_ |
| **a, b, c (Å)** | 45.82 74.75 74.80 | 46.04 74.64 74.88 | 45.84 74.41 74.69 |
| α, β, γ (˚) | 90 90 90 | 90 90 90 | 90 90 90 |
| Unique reflections | 40990 (2047) | 24175 (1670) | 34220 (1606) |
| Multiplicity | 6.5 (6.0) | 13.1 (12.8) | 6.6 (6.5) |
| Completeness (%) | 99.6 (98.6) | 99.6 (97.8) | 99.3 (95.8) |
| **I/σ(I)** | 16.5 (1.8) | 14.5 (1.7) | 11.2 (1.6) |
| Wilson B-factor | 15.49 | 18.35 | 20.18 |
| **R_merge_** | 0.059 (0.768) | 0.134 (1.962) | 0.056 (0.896) |
| **R_p.i.m._** | 0.037 (0.50) | 0.054 (0.816) | 0.035 (0.566) |
| **CC_1/2_** | 0.999 (0.842) | 0.999 (0.797) | 0.999 (0.852) |
| ***Data Refinement Statistics*** | | | |
| Resolution range (Å) | 37.38 - 1.51  (1.53 - 1.51) | 37.32 - 1.81  (1.85 - 1.81) | 39.02 - 1.6  (1.63 - 1.60) |
| R_work_ | 0.1674 (0.3033) | 0.1833 (0.2766) | 0.1712 (0.3022) |
| R_free_ | 0.1909 (0.3169) | 0.2198 (0.2927) | 0.1942 (0.3083) |
| RMS(bonds) (Å) | 0.007 | 0.008 | 0.007 |
| RMS(angles) (°) | 1.00 | 1.09 | 1.05 |
| Ramachandran favored (%) | 97.41 | 97.78 | 97.41 |
| Ramachandran allowed (%) | 1.85 | 2.22 | 2.22 |
| Ramachandran outliers (%) | 0.74 | 0.00 | 0.37 |
| Rotamer outliers (%) | 0.88 | 0.45 | 0.00 |
| Average B-factor | 22.73 | 26.70 | 27.61 |
