# Supplementary material for: Selenocyanate derived Se-incorporation into the nitrogenase Fe protein cluster
Source: eLife. 2022 Jul 29;11:e79311. doi: 10.7554/eLife.79311 (PMC9462850; doi:10.7554/eLife.79311)
Supplement: Supplementary file 6. [file elife-79311-supp6.docx]

Data collection and refinement statistics for Se-incorporated Fe protein crystals derived from *22 mM* KSeCN reaction in the *absence* of MoFe protein. Values in parentheses represent the highest resolution shell.

| *Data Processing Statistics* | | | | |
| --- | --- | --- | --- | --- |
| PDB ID | **7TQC** | **7TNE** | **7TQE** | **7TQF** |
| Wavelength (Å) | 12668 | 12668 | 12668 | 12668 |
| Resolution range (Å) | 45.60 - 1.53  (1.56 - 1.53) | 45.76 - 1.39  (1.42 - 1.39) | 45.88 - 1.59  (1.62 - 1.59) | 45.58 - 1.45  (1.48 - 1.45) |
| Space group | P22_1_2_1_ | P22_1_2_1_ | P22_1_2_1_ | P22_1_2_1_ |
| a, b, c (Å) | 45.60 74.45 74.76 | 45.73 74.40 74.98 | 45.90 74.67 74.75 | 45.60 74.17 74.69 |
| α, β, γ (˚) | 90 90 90 | 90 90 90 | 90 90 90 | 90 90 90 |
| Unique reflections | 39148 (1875) | 52125 (2621) | 35230 (1813) | 45611 (2363) |
| Multiplicity | 12.9 (12.2) | 12.9 (11.5) | 13.1 (12.9) | 12.9 (12.4) |
| Completeness (%) | 99.9 (99.1) | 99.8 (97.1) | 99.8 (99.5) | 99.9 (98.6) |
| I/σ(I) | 10.9 (1.9) | 14.2 (2.0) | 21.1 (2.0) | 19.0 (2.1) |
| Wilson B-factor | 15.76 | 16.95 | 19.54 | 17.55 |
| R_merge_ | 0.098 (0.884) | 0.077 (0.757) | 0.068 (1.405) | 0.064 (1.057) |
| R_p.i.m._ | 0.04 (0.382) | 0.031 (0.328) | 0.028 (0.583) | 0.026 (0.445) |
| CC_1/2_ | 0.999 (0.964) | 0.999 (0.933) | 1.00 (0.873) | 0.999 (0.917) |
| ***Data Refinement Statistics*** | | | | |
| Resolution range (Å) | 37.23 - 1.53  (1.55 - 1.53) | 37.49 - 1.39  (1.41 - 1.39) | 37.38 - 1.59  (1.61 - 1.59) | 38.84 - 1.45  (1.47 - 1.45) |
| R_work_ | 0.1637 (0.2660) | 0.1684 (0.2810) | 0.1731 (0.3066) | 0.1836 (0.3189) |
| R_free_ | 0.1917 (0.2881) | 0.1876 (0.2482) | 0.1990 (0.3374) | 0.2034 (0.3402) |
| RMS(bonds) (Å) | 0.007 | 0.006 | 0.006 | 0.006 |
| RMS(angles) (°) | 1.03 | 1.02 | 1.02 | 1.02 |
| Ramachandran favored (%) | 97.43 | 98.18 | 97.79 | 97.42 |
| Ramachandran allowed (%) | 2.21 | 1.46 | 1.85 | 2.58 |
| Ramachandran outliers (%) | 0.37 | 0.36 | 0.37 | 0.00 |
| Rotamer outliers (%) | 0.00 | 0.43 | 2.63 | 0.00 |
| Average B-factor | 22.50 | 24.02 | 27.51 | 25.38 |
