# Supplementary material for: Selenocyanate derived Se-incorporation into the nitrogenase Fe protein cluster
Source: eLife. 2022 Jul 29;11:e79311. doi: 10.7554/eLife.79311 (PMC9462850; doi:10.7554/eLife.79311)
Supplement: Supplementary file 7. [file elife-79311-supp7.docx]

Data collection and refinement statistics for Se-incorporated Fe protein crystals derived from *11 mM* KSeCN reaction in the *absence* of MoFe protein. Values in parentheses represent the highest resolution shell.

| *Data Processing Statistics* | | | |
| --- | --- | --- | --- |
| PDB ID | **7TPN** | **7TQH** | **7TQI** |
| Wavelength (Å) | 12668 | 12668 | 12668 |
| Resolution range (Å) | 46.01 - 1.38  (1.40 - 1.38) | 45.72 - 1.49  (1.52 - 1.49) | 45.86 - 1.47  (1.50 - 1.47) |
| Space group | P22_1_2_1_ | P22_1_2_1_ | P22_1_2_1_ |
| a, b, c (Å) | 46.0 74.63 75.14 | 45.74 74.68 74.92 | 45.79 74.68 75.18 |
| α, β, γ (˚) | 90 90 90 | 90 90 90 | 90 90 90 |
| Unique reflections | 53696 (2653) | 42696 (2237) | 44627 (2237) |
| Multiplicity | 13.0 (12.4) | 13.1 (12.4) | 13.1 (13.2) |
| Completeness (%) | 99.6 (98.4) | 99.9 (99.6) | 99.9 (98.9) |
| I/σ(I) | 13.7 (1.9) | 18.4 (1.8) | 16.6 (2.2) |
| Wilson B-factor | 15.22 | 18.03 | 17.59 |
| R_merge_ | 0.094 (1.669) | 0.071 (1.319) | 0.079 (1.211) |
| R_p.i.m._ | 0.039 (0.705) | 0.029 (0.562) | 0.032 (0.499) |
| CC_1/2_ | 0.999 (0.775) | 0.999 (0.835) | 0.999 (0.909) |
| ***Data Refinement Statistics*** | | | |
| Resolution range (Å) | 46.00 - 1.38  (1.40 - 1.38) | 39.01 - 1.49  (1.51 - 1.49) | 39.03 - 1.47  (1.49 - 1.47) |
| R_work_ | 0.1763 (0.3185) | 0.1875 (0.3059) | 0.1760 (0.2820) |
| R_free_ | 0.1973 (0.3345) | 0.2038 (0.2911) | 0.1977 (0.3175) |
| RMS(bonds) (Å) | 0.006 | 0.007 | 0.006 |
| RMS(angles) (°) | 1.07 | 1.16 | 1.09 |
| Ramachandran favored (%) | 97.79 | 97.03 | 98.15 |
| Ramachandran allowed (%) | 1.84 | 2.60 | 1.48 |
| Ramachandran outliers (%) | 0.37 | 0.37 | 0.37 |
| Rotamer outliers (%) | 0.00 | 0.44 | 0.43 |
| Average B-factor | 21.91 | 24.87 | 24.96 |
