# Supplementary material for: Selenocyanate derived Se-incorporation into the nitrogenase Fe protein cluster
Source: eLife. 2022 Jul 29;11:e79311. doi: 10.7554/eLife.79311 (PMC9462850; doi:10.7554/eLife.79311)
Supplement: Supplementary file 8. [file elife-79311-supp8.docx]

Data collection and refinement statistics for Se-incorporated Fe protein crystals derived from *1 mM* KSeCN reaction in the *absence* of MoFe protein. Values in parentheses represent the highest resolution shell.

| *Data Processing Statistics* | | | |
| --- | --- | --- | --- |
| PDB ID | **7TPO** | **7TQJ** | **7TQK** |
| Wavelength (Å) | 12668 | 12668 | 12668 |
| Resolution range (Å) | 45.85 - 1.351  (1.37 - 1.35) | 45.75 - 1.48  (1.51 - 1.48) | 39.05 - 1.48  (1.51 - 1.48) |
| Space group | P22_1_2_1_ | P22_1_2_1_ | P22_1_2_1_ |
| a, b, c (Å) | 45.88 74.76 75.14 | 45.79 74.31 74.74 | 45.75 74.30 74.66 |
| α, β, γ (˚) | 90 90 90 | 90 90 90 | 90 90 90 |
| Unique reflections | 57097 (2769) | 43295 (2260) | 43127 (2143) |
| Multiplicity | 13.2 (11.2) | 13.3 (12.1) | 13.2 (11.5) |
| Completeness (%) | 99.3 (98.8) | 100.00 (99.9) | 99.7 (98.3) |
| I/σ(I) | 9.0 (1.8) | 8.9 (1.7) | 10.3 (1.9) |
| Wilson B-factor | 14.47 | 17.21 | 20.30 |
| R_merge_ | 0.161 (0.890) | 0.172 (2.131) | 0.129 (1.990) |
| R_p.i.m._ | 0.064 (0.396) | 0.069 (0.921) | 0.052 (0.886) |
| CC_1/2_ | 0.987 (0.902) | 0.991 (0.813) | 0.994 (0.745) |
| ***Data Refinement Statistics*** | | | |
| Resolution range (Å) | 39.16 - 1.351  (1.37 - 1.35) | 37.16 - 1.48  (1.50 - 1.48) | 38.96 - 1.48  (1.50 - 1.48) |
| R_work_ | 0.1788 (0.2966) | 0.1785 (0.3349) | 0.1856 (0.3202) |
| R_free_ | 0.2032 (0.3130) | 0.2050 (0.3783) | 0.2028 (0.3642) |
| RMS(bonds) (Å) | 0.006 | 0.007 | 0.007 |
| RMS(angles) (°) | 1.07 | 1.11 | 1.08 |
| Ramachandran favored (%) | 98.15 | 98.15 | 97.42 |
| Ramachandran allowed (%) | 1.48 | 1.85 | 2.21 |
| Ramachandran outliers (%) | 0.37 | 0.00 | 0.37 |
| Rotamer outliers (%) | 0.00 | 0.00 | 0.44 |
| Average B-factor | 21.01 | 24.85 | 27.40 |
