# Supplementary material for: Selenocyanate derived Se-incorporation into the nitrogenase Fe protein cluster
Source: eLife. 2022 Jul 29;11:e79311. doi: 10.7554/eLife.79311 (PMC9462850; doi:10.7554/eLife.79311)
Supplement: Supplementary file 9. [file elife-79311-supp9.docx]

Data collection and refinement statistics for Fe protein crystal soaked with KSeCN.

| *Data Processing Statistics* | |
| --- | --- |
| PDB ID | **7TPV** |
| Wavelength (Å) | 12668 |
| Resolution range (Å) | 45.35 - 1.49  (1.52 - 1.49) |
| Space group | P22_1_2_1_ |
| a, b, c (Å) | 45.36 73.69 74.17 |
| α, β, γ (˚) | 90 90 90 |
| Unique reflections | 41143 (2448) |
| Multiplicity | 13.0 (12.4) |
| Completeness (%) | 99.4 (99.5) |
| I/σ(I) | 17.9 (2.2) |
| Wilson B-factor | 16.89 |
| R_merge_ | 0.071 (1.095) |
| R_p.i.m._ | 0.029 (0.462) |
| CC_1/2_ | 0.999 (0.877) |
| ***Data Refinement Statistics*** | |
| Resolution range (Å) | 38.70 - 1.49  (1.51 - 1.49) |
| R_work_ | 0.1748 (0.2996) |
| R_free_ | 0.1989 (0.2796) |
| RMS(bonds) (Å) | 0.006 |
| RMS(angles) (°) | 1.04 |
| Ramachandran favored (%) | 98.18 |
| Ramachandran allowed (%) | 1.82 |
| Ramachandran outliers (%) | 0.00 |
| Rotamer outliers (%) | 0.43 |
| Average B-factor | 24.86 |
